# Genetic Diversity of Sangihe Nutmeg (*Myristica fragrans* Houtt.) Based on Morphological and ISSR Markers

**DOI:** 10.1155/sci5/5568104

**Published:** 2024-12-28

**Authors:** Tanti Agustina, Abdul Razaq Chasani, Budi Setiadi Daryono, Muhammad Said Rifqi

**Affiliations:** ^1^Laboratory of Plant Systematics, Department of Biology, Faculty of Biology, Universitas Gadjah Mada, Sekip Utara Street, Sleman, Yogyakarta 55281, Indonesia; ^2^Laboratory Genetics and Breeding, Department of Biology, Faculty of Biology, Universitas Gadjah Mada, Sekip Utara Street, Sleman, Yogyakarta 55281, Indonesia

**Keywords:** inter simple sequence repeat (ISSR), morphology, *Myristica fragrans*, phenetic relationship, Sangihe

## Abstract

Sangihe nutmeg is an important crop because of its usefulness in the pharmacology, spices and cosmetics industries. Sangihe is the oldest active subduction zone island in the Indonesia–Philippines region, where frequent tectonic earthquakes and the geographic and reproductive isolation of Sangihe nutmeg occur. This isolation results in adaptation and speciation because of increasing variability. Using morphological and molecular approaches, we aimed to assess the genetic variety of Sangihe nutmeg based on morphological and intersimple sequence repeat (ISSR) markers. In total, 31 morphological characteristics were examined, and molecular data of ISSR markers using five primers were analysed based on numerical taxonomy using the unweighted pair group method with arithmetic mean (UPGMA) and principal component analysis (PCA) methods. Results showed five major groups of Sangihe nutmeg based on seed variation, that is, thick round, thin round, thick oval, thin oval, and twin seeds, whereas the presence of variation in molecular characters was indicated by DNA polymorphisms between 0% and 33.33%. The phenetic relationships within Sangihe nutmeg, based on morphological and ISSR markers, exhibit two groups with different member compositions. The major morphological characteristics influencing the phenetic relationship pattern were tree shape, leaf shape, fruit shape, number of fractions when ripe, fruit size index, number of fruit indentations, indentation, aryl density, seed shell, and number of seeds.

## 1. Introduction

Sangihe nutmeg (*Myristica fragrans* Houtt.) is one of the major sources of spices as it contains triterpene essential oils, aromatic compounds and various types of phenolic compounds that are potential medicines [1, 2]. Nutmeg has many uses ranging from culinary to medicinal, especially in food flavouring essential oil applications and traditional medicines [3, 4]. It can help lower blood pressure, soothe stomach aches and stop diarrhoea, as well as (at low doses) detoxify the body and stimulate the brain [5]. Indonesia dominates the production and export of nutmeg globally [6, 7]. Considerably, nutmeg originated from Indonesia, especially from Maluku [8]. Although it originated from Maluku, the largest production of nutmeg in Indonesia is on Sangihe Island presently. Sangihe Island is surrounded by oceans and is the oldest active subduction zone in the Indonesia–Philippines region where frequent tectonic earthquakes occur [9]. This isolation causes geographic and reproductive isolation in Sangihe nutmeg.

Geographic and reproductive isolation on Sangihe Island facilitates adaptation and speciation, thereby increasing variation, including that of nutmeg [10, 11]. Information on the biodiversity of nutmeg in Indonesia is limited [12, 13], with the lack of molecular identification being one reason. The barcode discrimination ability of *matK* [14] and *rbcL* [15] is low and cannot distinguish between species in the genus Myristica.

The variation in morphological and agronomic characteristics of nutmeg is high [16]. Agronomic characters are morphological characteristics beneficial for pomologists, for example, fruit diameter index, seed diameter index, fruit weight, seed weight and mace weight. Additionally, the chemical composition of nutmeg might vary with environment, variety and geographical location [3, 17]. Variations in morphological characteristics and chemical composition of nutmeg supported by typical growing locations in Sangihe Island suggest high variability in Sangihe nutmeg.

Information on character variation and phenetic relationships in nutmeg on Sangihe Island is limited. In order to reveal information on genetic variation in Sangihe nutmeg, it can be done using intersimple sequence repeat (ISSR) markers, which are genomic regions flanked by microsatellite sequences. Amplification products in this region can be used for a dominant multilocus marker system to study genetic variation in various organisms [18]. According to Poyraz [19], ISSR markers are very appropriate for use in the study of the genetic variation of a species with high genetic diversity among populations. ISSR markers are considered to be more sensitive to detecting genetic differences between populations in the same habitat. Research on this subject is necessary to determine the genetic diversity of an important crop. Diversity in plant genetic resources can provide an opportunity to develop improved and new cultivars of crops according to desired characteristics, such as farmer- (yield potential and large seeds) and breeder-preferred traits (resistance to pests and diseases and photosensitivity) [20].

The objective of the present study was to assess the genetic variety of Sangihe nutmeg based on morphological and ISSR markers. The results of this study are expected to improve scientific information and the genetic improvement of *Myristica fragrans* on Sangihe Island. The obtained data will be a source of information that underlies the further conservation and breeding programmes for high-yielding nutmeg plant varieties from Sangihe Island.

## 2. Materials and Methods

### 2.1. Sample Collection and Morphological Observations

**Figure 1:**
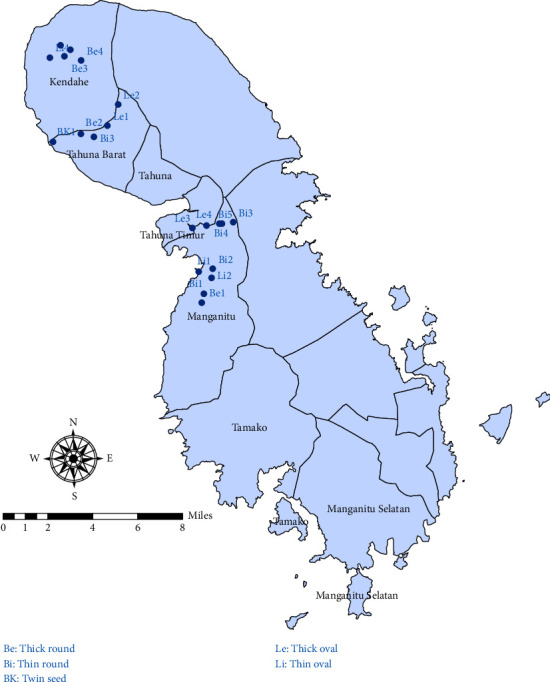
Distribution of sampling locations (● = sampling locations).

Samples were collected from four locations that indicated a high population of Sangihe nutmeg (see Figure 1 and Table 1). From the selected location, 20 individuals of nutmeg were obtained and used as operational taxonomic units (OTUs). In total, 31 morphological characteristics (see Table 2) from five types of Sangihe nutmeg were observed and measured from each location, including one character of the habitus, four characters of leaves, 16 characters of fruits, and 10 characters of seeds. Morphological characters were used to evaluate consist of qualitative (tree shape, leaf shape, fruit shape, fruit sap colour, etc.) and quantitative characters (leaf stalk length, fruit diameter, number of seed, etc.). The morphological characteristics of nutmeg were examined due to their significant taxonomic potential [21] and high diversity. For molecular analysis, leaf samples from the same order of leaf branches were collected.

### 2.2. Molecular Observation

**Figure 2:**
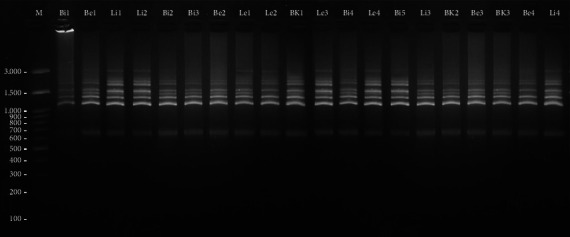
Electrophoresis results of 20 nutmeg samples from 4 sub-districts in Sangihe Island using UBC 852A primers. Notes: M: marker Geneaid 100 bp DNA ladder. Bi: thin round; Be: thick round; Li: thin oval; Le: thick oval; BK: twin seed.

Molecular character observation consisted of DNA isolation, a quality test of isolated DNA using electrophoresis, amplification by ISSR-PCR using 8 ISSR primers (see Table 3) and qualitative test of amplification results using electrophoresis. Genomic DNA was isolated using the Geneaid extraction kit (GR100) with a modified protocol. The quality test of isolated DNA was conducted on a 2% agarose gel and documented using a UV–Vis transilluminator. The PCR reaction mixture contained 12.5 μL My Taq Hs Red Mix, 1 μL Primer ISSR, 2 μL DNA Template and 8.5 μL nuclease-free water with 25 μL final volume. The PCR programme was designed as follows: initial denaturation of 4 min at 94°C, followed by 35 cycles of denaturation for 35 s at 94°C, primers annealing for 1 min at 53°C and extension for 10 min at 72°C, followed by a final extension for 10 min at 72°C. Amplified products were size-separated on 2% agarose gel and documented using a UV–Vis transilluminator. The result of ISSR amplification of primer UBC 852A for 20 Sangihe nutmeg is shown in Figure 2.

### 2.3. Data Analysis

Descriptive and statistical data analyses were conducted. Descriptive analysis involved a detailed description of the characteristics of each group in the dendrogram. Phenetic relationships were analysed using UPGMA cluster analysis and PCA in the Multivariate Statistical Package 3.1 programme [22]. The UPGMA algorithm and the Jaccard coefficient were used as references in constructing the dendrogram, whereas PCA showed the eigenvalues of each cluster formation. The results of the variability analysis of Sangihe nutmeg are presented as a list of types, and phenetic relationship patterns are indicated as a phenogram for clustering analysis and scatter diagrams for ordination. Morphological diversity level of Sangihe nutmeg was analysed with Shannon–Wiener index equation (1), which is as follows:

(1) $$\begin{array}{c} H^{\prime }=-\overset{s}{\underset{t=s}{\sum }}pi\text{ In }pi. \end{array}$$

Description: *H*′: Shannon–Wiener index.

*pi*: *ni*/*N*.

*ni*: Total individual of each *i-*th characters.

*N*: Total number of observed plant samples.

There are three classifications of diversity levels based on the Shannon–Wiener diversity index: (i) *H*′ < 1, indicating low diversity, (ii) 1 < *H*′ < 3, indicating medium diversity and (iii) *H*′ > 3, indicating high diversity [23]. The genetic structure of *M. fragrans* based on ISSR markers was analysed with the Structure 2.3.4 software [24]. The number of clusters (*K*) was set from 1 to 8 (10 times for each cluster) for the assessment by the Structure software. The length of the burn-in period was 10,000, and the number of MCMC was 100,000. In order to detect the optimum value of K, we use the Structure Selector software (https://lmme.ac.cn/StructureSelector/).

## 3. Results

### 3.1. Morphological Variation

*Myristica fragrans* is a 9–15 m tall, dioecious tree, with a red gummy and sticky texture. This result is different from Wu's, Raven and Hong [25] description of *M. fragrans* as a small tree with a maximum height of 10 m. Similarly, Sarma, Babu and Aziz [26] described *M. fragrans* as having an average height of 10–20 m with spreading branches. This difference in tree height data is probably because the nutmeg on the Sangihe Island is 100 s of years old, so they can reach a height of 15 m.

A single leaf stipule has a distinctive aroma, green, alternate and ovate to oblanceolate. The upper and lower surfaces of leaves are dark and pale green, petiole 1.20–2.40 cm, apiculate tip, cuneate base, entire, width 4.10–5.40 cm and length 12.70–18.80 cm. The characteristics of the leaf shape are different from those of Wu, Raven and Hong [25], who stated that the leaf shape of *M. fragrans* is elliptic or elliptic-lanceolate, but it is in line with Ross' description [27], who stated that the leaf shape of *M. fragrans* is ovate-elliptical. Moreover, the colour and surface of the leaves were in accordance with Kumari, Kaurav and Chaudhary [28], who stated that the surface of the leaves of *M. fragrans* was glossy, with the upper and lower surfaces of the leaves being green and pale green.

**Figure 3:**
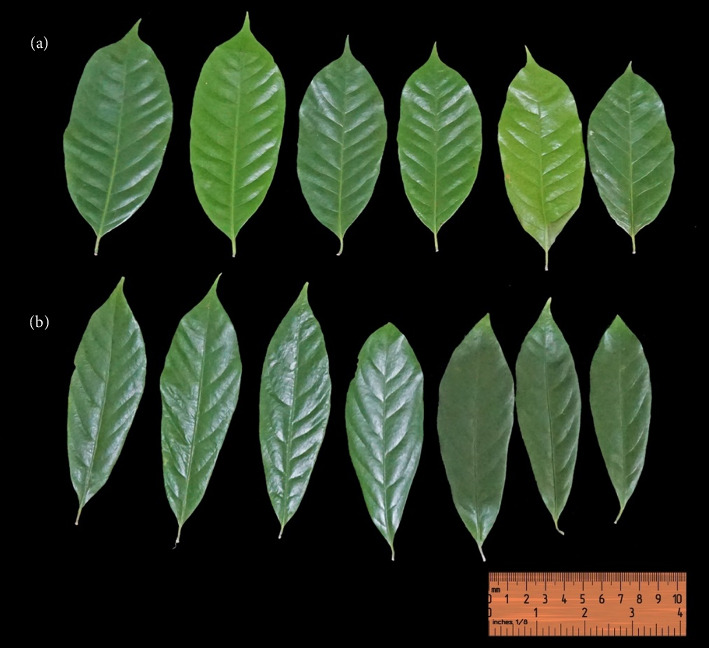
Variation in leaf morphology of *Myristica fragrans*: (a) ovate-shaped leaves; (b) leaves are oblanceolate in shape.

The leaf shape varies from oval to oblanceolate. The leaf shape variations found in this study are similar to those found by Hetharie et al. [29], who examined the morphology of nutmeg in Liliboi Village, Ambon Island. The shape of nutmeg leaves varies from elliptical to oblong elliptical. Reportedly, round fruit-type nutmeg has oval-shaped leaves, whereas oval fruit types tend to have oblanceolate leaves (see Figure 3).

**Figure 4:**
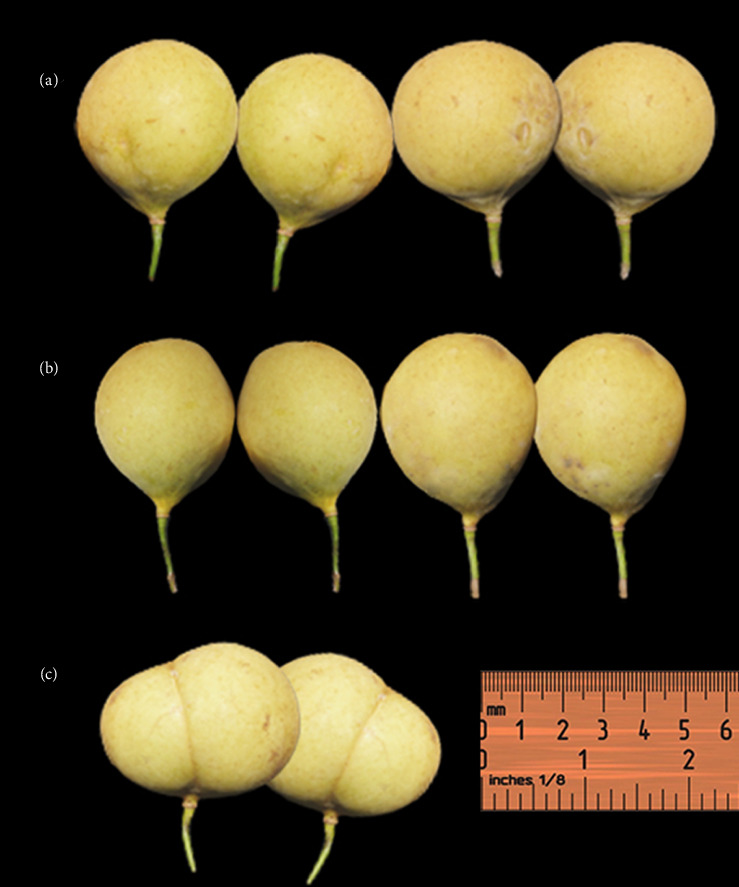
Morphological variation of *Myristica fragrans* fruit shape: (a) round fruit, (b) fruit oval and (c) peaches.

**Figure 5:**
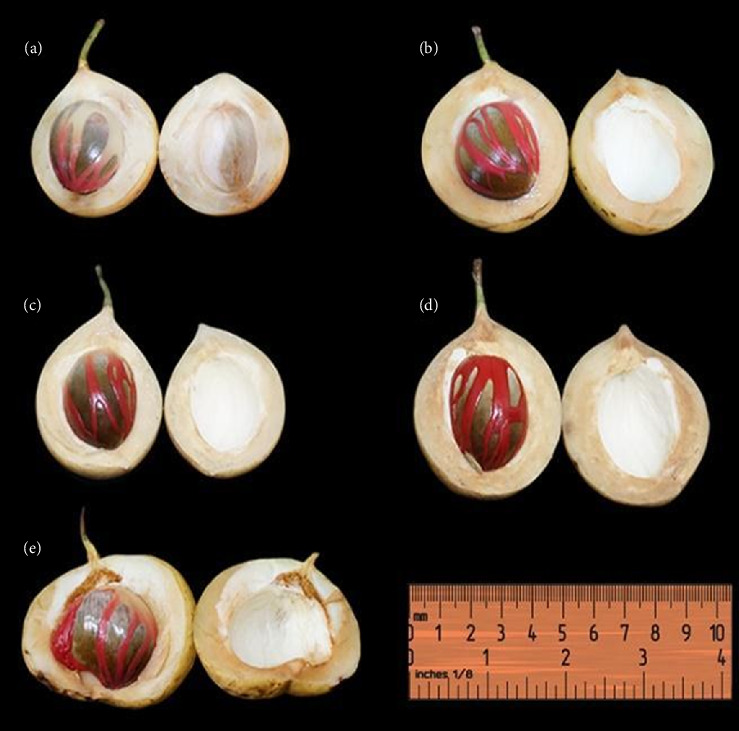
Morphological variations in epicarp thickness and seed shape of nutmeg: (a) round fruit, round seeds (thin epicarp), (b) round fruit, round seeds (thick epicarp), (c) oval fruit, oval seeds (thin epicarp), (d) oval fruit, oval seeds (thick epicarp), (e) round lobed fruit, two oval or one lobed seed.

The shape of the Sangihe nutmeg also varies; there are round, oval and round peach shapes [30] (see Figure 4). The oval and round fruit splits into two parts with one seed, whereas the peach-shaped fruit splits into four parts with one to two seeds. The nutmeg has a clear to white sap with 4.70–6.20 cm length and 3.80–5.50 cm diameter. The exocarp is yellow, the epicarp is 0.80–1.3 cm and the mesocarp is pale yellow (0.60–1.00 cm) (see Figure 5). The end of the fruit is rounded with a tapered base; the pedicel is 1.20–2.40 cm. The seed coat is covered by red aril, with 1–2 seeds, glossy, dark brown seed shell, 1.80–3.00 cm seed diameter, and 3.00–3.50 cm seed length.

### 3.2. Clustering and Diversity Based on Morphological Characteristics

**Figure 6:**
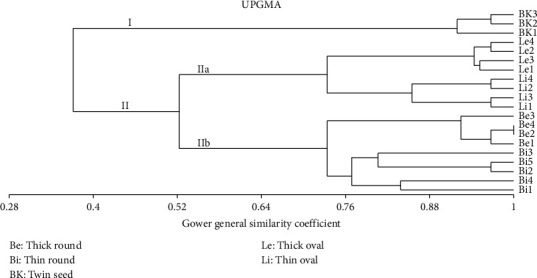
UPGMA dendrogram of Sangihe nutmeg based on the Gower's general similarity coefficient.

**Figure 7:**
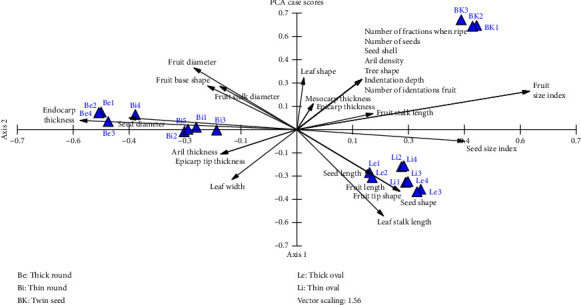
PCA scatter plot of Sangihe nutmeg (*Myristica fragrans*).

The UPGMA (see Figure 6) and PCA analyses (see Figure 7) based on the morphology of Sangihe nutmeg resulted in two main groups. Group I consisted of twin-seeded nutmeg, and Group II consisted of oval and round nutmeg. Additionally, Group II consists of two subgroups that is IIa (oval seed) and IIb (round seed).

The similarity value of *M. fragrans* based on the dendrogram is 37.1%–100%, with clusters I and II having a similarity value of 37.1%. Cluster I comprised three nutmegs with twin seeds. This group had similar characteristics, including leaf shape, number of seeds, number of fruit fragments when ripe, fruit size index, fruit stalk length and mesocarp thickness. Cluster II included nine round and eight oval nutmegs; cluster IIa had a similar tendency in the index of seed size, seed length, seed shape, fruit length and leaf stalk length. Cluster IIb had similar characteristics concerning fruit diameter, fruit stalk diameter, seed diameter, endocarp thickness, aril thickness and leaf width.

Based on PCA of 31 morphological characteristics (see Table 2), the total eigenvalue on axis one is 2.856 and that on axis two is 2.26 (see Table 4). In this study, the axes used as a reference are axes 1 and 2 because they have high eigenvalues. According to Jeffers [31], a component with an eigenvalue of 1 has a significant value, and according to Susandarini et al. [32], a character can influence if it has a loading variable of 0.2. According to the PCA variable loadings result (see Table 5), out of 31 characters analysed, 10 characters that most influenced the grouping pattern were tree shape, leaf shape, fruit shape, number of fractions when ripe, fruit size index, number of fruit indentations, indentation, aryl density, seed shell and number of seeds.

The morphological diversity of Sangihe nutmeg was analysed with the Shannon–Wiener diversity index shown in Table 6. This result of the analysis shows that the diversity of nutmeg is in the moderate category, with an average Shannon index value of 2.971. Ortis-Burgos [33] explains that the main objectives of the Shannon–Wiener diversity index are to obtain a quantitative estimate of biological variability that can be used to compare biological entities in space and time. The index considers two different aspects that contribute to the concept of diversity in a community, specifically species richness and evenness.

### 3.3. Molecular Clustering and Genetic Structure Based on ISSR Markers

The molecular characteristics were analysed using ISSR markers. The PCR results showed that the eight ISSR markers could amplify nutmeg DNA, resulting in 92 fragments (see Figures S1–S8 in the Supporting Information for comprehensive image analysis). All primers produced various percentages of DNA polymorphisms ranging from 0.00% to 33.33% (see Table 7).

**Figure 8:**
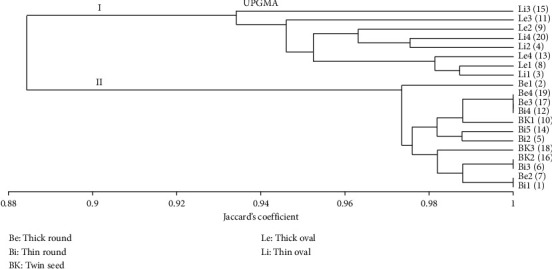
Dendrogram obtained from molecular characters from 20 accessions of nutmeg (*Myristica fragrans*) with UPGMA based on Jaccard's coefficient.

Based on the dendrogram, as the results of molecular analysis on *M. fragrans* based on ISSR markers showed (see Figure 8), two clusters formed divided into a group of oval nutmeg seeds and a combination of round seed and twin-seed nutmeg groups. This cluster revealed that the resulting grouping was based on the shape of the fruit. Cluster I consisted of eight collections of oval nuts with a 93% similarity value; cluster II consisted of round nuts and twin seeds with a 97% similarity value. The high similarity value between *M. fragrans* samples indicates a low level of genetic diversity. These results are similar to those of Tallei and Kolondam [14], who reported that there is no molecular diversity of *M. fragrans* based on the *matK* gene sequence, suggesting that the molecular diversity among species within the genus *Myristica* is low. Chen et al. [34] also stated a low level of polymorphism in *Nelumbo nucifera* by 55.61% and polymorphism in *Ctenopharyngodon idella* by 39.61% [35]. Another study using ISSR markers on *Triticum aestivum* reported that the polymorphism between the wheat genotypes studied was low, with the highest polymorphism value of 40.86% [36].

**Figure 9:**
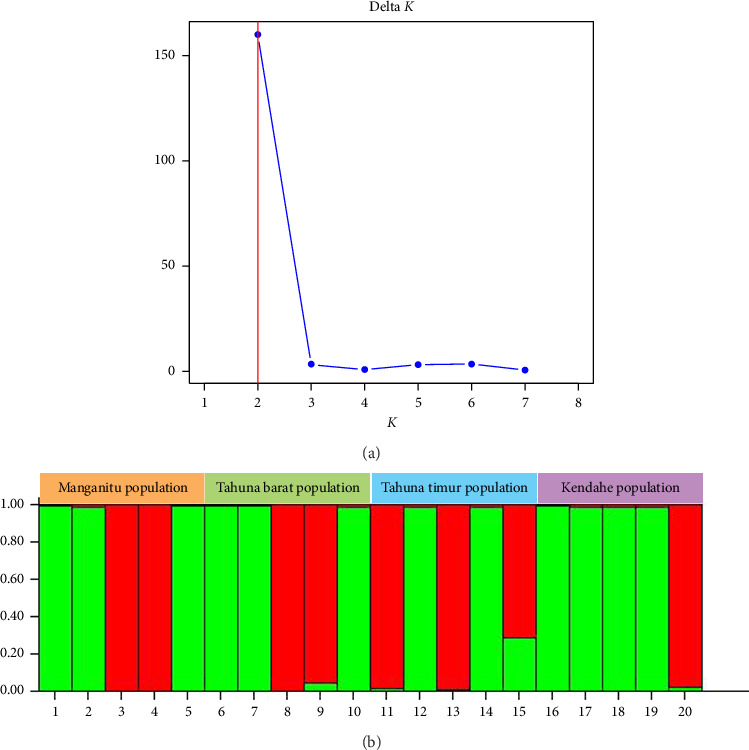
Population structure analysis of 20 Sangihe nutmeg samples, (a) best estimated delta *K* value, and (b) estimated population structure for *K* = 2. The red and green colours represent genetic groups designed by structure selector.

The Bayesian model-based population analysis of the 20 Sangihe nutmeg samples showed two distinct groupings (see Figure 9(b)). This result is shown by the delta *K* (Δ*K*) analysis using Structure Selector, which shows a sharp peak at *K* = 2 (see Figure 9(a)).

## 4. Discussion

The results of the analysis indicated by morphological and molecular markers showed different grouping patterns. According to morphological markers, nutmeg with a twin-seed type is grouped as paraphyletic by forming a cluster separated from the group of nutmeg with oval and round seed types. Based on ISSR molecular markers, nutmeg with a twin and a round seed type was grouped as monophyletic in one cluster, while nutmeg with an oval seed type formed a separate cluster. Differences in the formation of phenogram groupings can result from differences in the expression of a key gene, which are caused by environmental influences [37].

The separation of the two main clusters in the UPGMA clustering of Sangihe nutmeg using ISSR markers has a significant correspondence with the results of the Structure analysis. Genetic structure map detected the formation of two subgroups (Δ*K* = 2), which corresponded to cluster formation. Cluster I has more genetic components than Cluster II, as shown in green and red colours. The Kendahe population is the population with the lowest number of polymorphic loci, mostly formed from one genetic component (> 80%), whereas the Tahuna Timur population is the population that has the highest number of polymorphic loci, which are formed from two genetic components (> 46%).

Analysis of genetic diversity in Sangihe nutmeg using morphological characters shows moderate diversity with a Shannon index value of 2.971. Moderate diversity can be interpreted as meaning that the condition of the plant population community was stable regardless of interspecies competition related to food and space [38]. However, different results were shown in the results of the ISSR marker fragment analysis. The low percentage of DNA fragment polymorphism indicates that the level of genetic diversity in Sangihe nutmeg is low. Differences in morphological character diversity with molecular traits can be attributed to plasticity in the phenotype of nutmeg plants. Plasticity is the ability of plants to change their phenotype in response to the environment. This response is needed for plants to adapt to rapid climate change [39]. However, this genetic diversity does not reflect taxonomic grouping. Nevertheless, the analysis results of the genetic diversity in the Sangihe nutmeg can provide important initial information for nutmeg breeding programmes and support the results of morphological characterisation.

Apart from morphological characters, efforts to reveal the variability and relationships of Sangihe nutmeg need to be carried out using a molecular approach. Molecular analysis suitable for detecting intraspecies polymorphisms within *M. fragrans* is the ISSR method [40] because the ISSR marker has high efficiency in producing polymorphisms among varieties that have close relationships [41]. The results of this study successfully reveal the variability and phenetic relationship of *M. fragrans*; therefore, it will potentially direct the policy of the cultivation and development of Sangihe nutmeg, including conservation efforts and the development of new varieties in the future.

According to Campos et al. [42], the analysis of morphological characteristics to determine certain positions at the taxonomic level is insufficient and needs to be complemented by other methods to strengthen the relationships. The dendrogram of the relationship based on the morphological characteristics of *M. fragrans* showed two cluster formations. Cluster I consisted of the nutmeg type with twin seeds, and cluster II consisted of oval nutmeg. Cluster II formed two subclusters: cluster IIa consisting of oval fruit-type nutmeg, and cluster IIb consisting of round fruit-type nutmeg. The dendrogram of relationships based on molecular characteristics formed two clusters consisting of oval and round fruit-type nutmegs, in addition to twin seed-type nutmeg.

The variability or diversity of a plant can be identified by phenotype and genotype. Phenotypic variation in this study was observed based on morphological and genotypic characteristics based on genetics [43]. Phenotypic variation is the result of genetic expression. Genetic variation is passed down to the next generation and can be caused by mutations, gene recombination, migration, selection, genetic drift and plant breeding programmes especially in cultivated plants [44, 45]. However, genetic characteristics are more resistant to environmental conditions than morphological characteristics [43]. The similarity of traits included in the dendrogram can be used as the basis for phenetic grouping because, according to the Adansonian principle [46], the more characters used, the stronger the taxonomic data obtained.

This difference in cluster formation is common. There are several studies on various species, such as plums [47], apples [48], pistachios [49] and grapevines [50], suggesting that there is a negative correlation between morphological and molecular data. Based on Zhang et al. [51], this correlation needs not be positive. However, genetic relationships observed using molecular markers can provide information about the history of cultivar formation but do not reflect what can be observed directly about agronomic traits [52].

## 5. Conclusions

The morphological characteristics of *M. fragrans* on Sangihe Island varied, consisting of bears with thick round, thin round, thick oval, thin oval and twin-seed nutmegs. The genotypic variation of nutmeg on Sangihe Island is low, as indicated by the value of DNA fingerprint polymorphism based on ISSR of 0.00%–33.33%. The phenetic relationship of *M. fragrans* on Sangihe Island based on morphological markers and ISSR markers formed two clusters with different group compositions. The morphological characteristics that influenced the phenetic relationship pattern of nutmeg are tree shape, leaf shape, fruit shape, number of fractions when ripe, fruit size index, number of fruit indentations, indentation, aryl density, seed shell and number of seeds.

## Figures and Tables

**Table 1:** Sangihe nutmeg (*Myristica fragrans*) type and sites collection.

Nutmeg type	Sample code	Districts	Coordinate site
Thin round 1	Bi1	Manganitu	3°32′52.5″N 125°30′48.3″E
Thick round 1	Be1	Manganitu	3°32′32.0″N 125°30′43.9″E
Thin oval 1	Li1	Manganitu	3°33′43.8″N 125°30′34.7″E
Thin oval 2	Li2	Manganitu	3°33′27.3″N 125°31′04.9″E
Thin round 2	Bi2	Manganitu	3°33′49.6″N 125°31′08.5″E
Thin round 3	Bi3	Tahuna Barat	3°38′54.8″N 125°26′32.6″E
Thick round 2	Be2	Tahuna Barat	3°39′04.2″N 125°26′03.0″E
Thick oval 1	Le1	Tahuna Barat	3°39′23.2″N 125°27′04.3″E
Thick oval 2	Le2	Tahuna Barat	3°40′13.7″N 125°27′28.3″E
Twin seed 1	BK1	Tahuna Barat	3°38′45.4″N 125°24′57.1″E
Thick oval 3	Le3	Tahuna Timur	3°35′22.9″N 125°30′20.7″E
Thin round 4	Bi4	Tahuna Timur	3°35′29.4″N 125°30′53.8″E
Thick oval 4	Le4	Tahuna Timur	3°35′34.3″N 125°31′22.6″E
Thin round 5	Bi5	Tahuna Timur	3°35′34.8″N 125°31′28.0″E
Thin oval 3	Bi3	Tahuna Timur	3°35′39.1″N 125°31′55.4″E
Twin seed 2	BK2	Kendahe	3°42′27.6″N 125°25′15.4″E
Thick round 3	Be3	Kendahe	3°42′04.8″N 125°25′24.0″E
Twin seed 3	BK3	Kendahe	3°42′19.3″N 125°25′38.8″E
Thick round 4	Be4	Kendahe	3°41′53.7″N 125°26′03.3″E
Thin oval 4	Li4	Kendahe	3°42′00.5″N 125°24′51.6″E

**Table 2:** List of morphological characters of *Myristica fragrans* based on the performed analysis.

Character	Character identifiers and numeric codes
Tree shape	Cone; widened in the middle
Leaf shape	Oblanceolate; ovate
Leaf stalk length (cm)	1; 1.1; 1.2
Leaf width (cm)	4.10–4.75; 4.75–5.40
Leaf length (cm)	12.70–15.75; 15.75–18.80
Fruit shape	Fruit; oval; peach
Number of fractions when ripe	2; 4
Fruit sap colour	Transparent; white; clear whiteness
Fruit length (cm)	4.70–5.45; 5.45–6.20
Fruit diameter (cm)	3.80–4.65; 4.65–5.50
Fruit size index	1:1; 3:2; 2:3
Number of indentations fruit	2; 4
Indentation depth	Low; currently
Epicarp thickness (cm)	0.80–1.05; 1.05–1.30
Mesocarp thickness (cm)	0.60–0.80; 0.80–1.00
Endocarp thickness (cm)	0.1; 0.15; 0.2
Epicarp tip thickness	Thinning; not thinning
Fruit tip shape	Rounded; slightly rounded
Fruit base shape	Tapered; rounded
Fruit stalk diameter (cm)	0.10–0.20; 1.80–2.40
Fruit stalk length (cm)	1.20–1.80; 1.80–2.40
Aril colour	Red; dark red
Aril thickness (cm)	0.05; 0.1
Aril density	Currently; seldom
Seed shell	Full; not full
Seed coat colour	Brown; dark brown
Number of seeds	1; 2
Seed shape	Round; oval
Seed diameter (cm)	1.80–2.40; 2.40–3.00
Seed length (cm)	3.00–3.25; 3.25–3.50
Seed size index	1:1; 2:3

**Table 3:** ISSR primer sequence.

Primer name	Primer sequence
UBC 852A	5′-GTGTGTGTGTGTGTGTCC-3′
UBC 857B	5′-ACACACACACACACACTG-3′
UBC 858	5′-TGTGTGTGTGTGTGTGRT-3′
UBC 807	5′-AGAGAGAGAGAGAGAGT-3′
UBC 834A	5′-AGAGAGAGAGAGAGAGCT-3′
UBC 840B	5′-GAGAGAGAGAGAGAGATT-3′
UBC 842B	5′-GAGAGAGAGAGAGAGATG-3′
UBC 810	5′-GAGAGAGAGAGAGAGAT-3′

**Table 4:** Eigenvalues principal component analysis results.

	Axis 1	Axis 2
Eigen values	**2.856**	**2.26**
Percentage	33.053	26.162
Cum. Percentage	33.053	59.215

*Note:* The bold values indicate the significance of eigenvalues in determining a component.

**Table 5:** Variable loadings results of principal component analysis of Sangihe nutmeg (*Myristica fragrans*).

PCA variable loadings	Axis 1	Axis 2
Tree shape	0.112	**0.207**
Leaf shape	0.012	**0.215**
Leaf stalk length	0.15	−0.358
Leaf width	−0.112	−0.207
Leaf length	0.03	−0.069
Fruit shape	**0.402**	0.159
Number of fractions when ripe	0.112	**0.207**
Fruit sap colour	−0.076	0.078
Fruit length	0.178	−0.256
Fruit diameter	−0.178	0.256
Fruit size index	**0.402**	0.159
Number of indentation fruit	0.112	**0.207**
Indentation depth	0.112	**0.207**
Epicarp thickness	0.028	0.107
Mesocarp thickness	0.028	0.107
Endocarp thickness	−0.374	0.038
Epicarp tip thickness	−0.131	−0.102
Fruit tip shape	0.178	−0.256
Fruit base shape	−0.154	0.181
Fruit stalk diameter	−0.133	0.18
Fruit stalk length	0.132	0.066
Aril colour	0.044	0.067
Aril thickness	−0.131	−0.102
Aril density	0.112	**0.207**
Seed shell	0.112	**0.207**
Seed coat colour	−0.026	−0.021
Number of seeds	0.112	**0.207**
Seed shape	0.178	−0.256
Seed diameter	−0.29	0.048
Seed length	0.13	−0.187
Seed size index	0.29	−0.048

*Note:* The bold values as variable loadings indicate morphological characters that significantly influence the clustering.

**Table 6:** Morphological diversity based on Shannon–Wiener index (H′).

Sample	Index	Evenness	Num. Spec
Tree shape	2.979	0.994	20
Leaf shape	2.970	0.991	20
Leaf stalk length	2.948	0.984	20
Leaf width	2.985	0.996	20
Leaf length	2.979	0.994	20
Fruit shape	2.959	0.988	20
Number of fractions when ripe	2.979	0.994	20
Fruit sap colour	2.960	0.988	20
Fruit length	2.969	0.991	20
Fruit diameter	2.972	0.992	20
Fruit size index	2.959	0.988	20
Number of indentation fruit	2.979	0.994	20
Indentation depth	2.979	0.994	20
Epicarp thickness	2.971	0.992	20
Mesocarp thickness	2.971	0.992	20
Endocarp thickness	2.944	0.983	20
Epicarp tip thickness	2.970	0.991	20
Fruit tip shape	2.969	0.991	20
Fruit base shape	2.969	0.991	20
Fruit stalk diameter	2.969	0.991	20
Fruit stalk length	2.970	0.991	20
Aril colour	2.979	0.994	20
Aril thickness	2.970	0.991	20
Aril density	2.979	0.994	20
Seed shell	2.979	0.994	20
Seed coat colour	2.979	0.994	20
Number of seeds	2.979	0.994	20
Seed shape	2.969	0.991	20
Seed diameter	2.969	0.991	20
Seed length	2.971	0.992	20
Seed size index	2.971	0.992	20
Average	2.971	0.992	20

**Table 7:** Profile of *Myristica fragrans* DNA amplification results using 8 ISSR primers.

Primer	Fragment (bp)	Total number of bands	Polymorphic bands	Polymorphic percentage (%)
UBC 852A	700–3.000	9	2	22.22
UBC 857B	550–3.000	9	3	33.33
UBC 858	550–3.000	11	2	18.18
UBC 807	900–2.500	9	0	0.00
UBC 434A	750–2.500	10	2	20.00
UBC 840B	400–3.000	14	2	14.28
UBC 842B	350–2.900	15	3	20.00
UBC 810	290–3.000	15	1	6.00

## Data Availability

All data have been included in the manuscript.

## Supporting Information

Supporting Information consisting of images of electrophoresis results on Sangihe nutmeg using 8 types of ISSR markers (Figures S1–S8).
