# Supplementary material for: Genetic Diversity of Sangihe Nutmeg (Myristica fragrans Houtt.) Based on Morphological and ISSR Markers
Source: Scientifica (Cairo). 2024 Dec 28;2024:5568104. doi: 10.1155/sci5/5568104 (PMC11699992; doi:10.1155/sci5/5568104)
Supplement: Supporting Information — Additional supporting information can be found online in the Supporting Information section. [file 5568104.f1.docx]

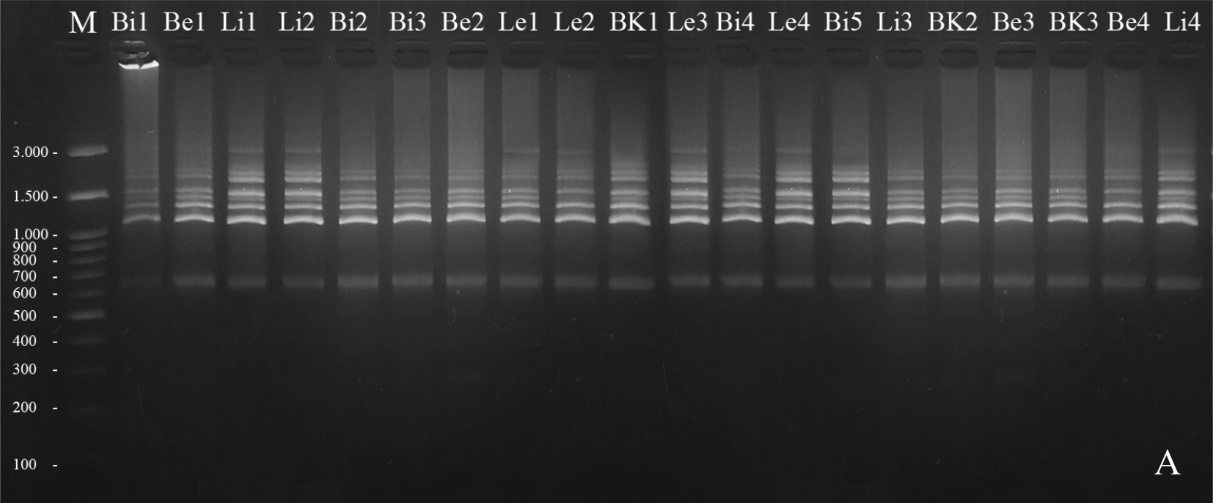


Figure S1: Electrophoresis results of 20 nutmeg samples from 4 sub-districts in Sangihe Island using UBC 852A primers. Notes: M: Marker Geneaid 100bp DNA Ladder. Bi: Thin round; Be: Thick round; Li: Thin oval; Le: Thick oval; BK: Twin seed.


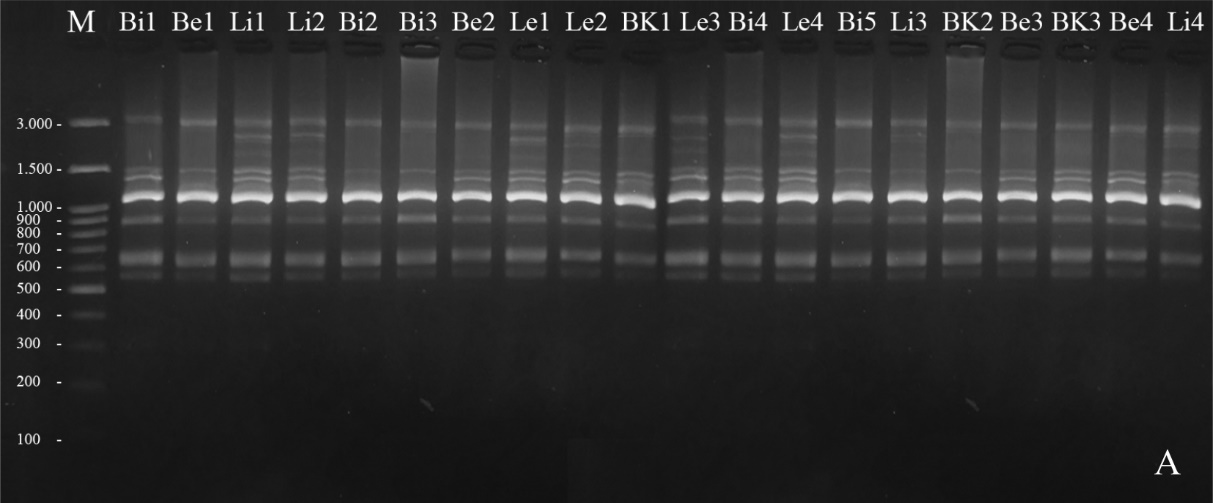


Figure S2: Electrophoresis results of 20 nutmeg samples from 4 sub-districts in Sangihe Island using UBC 857B primers. Notes: M: Geneaid 100 bp DNA Ladder marker. Bi: Thin round; Be: Thick round; Li: Thin oval; Le: Thick oval; BK: Twin seed.


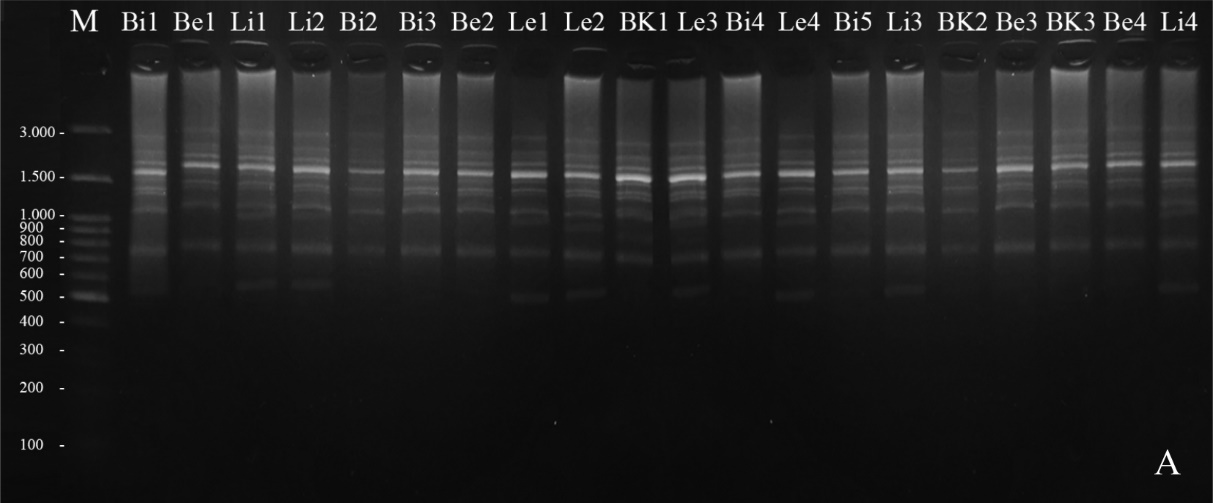


Figure S3: Electrophoresis results of 20 nutmeg samples from 4 sub-districts in Sangihe Island using UBC 858 primers. Notes: M: Geneaid 100 bp DNA Ladder marker. Bi: Thin round; Be: Thick round; Li: Thin oval; Le: Thick oval; BK: Twin seed.


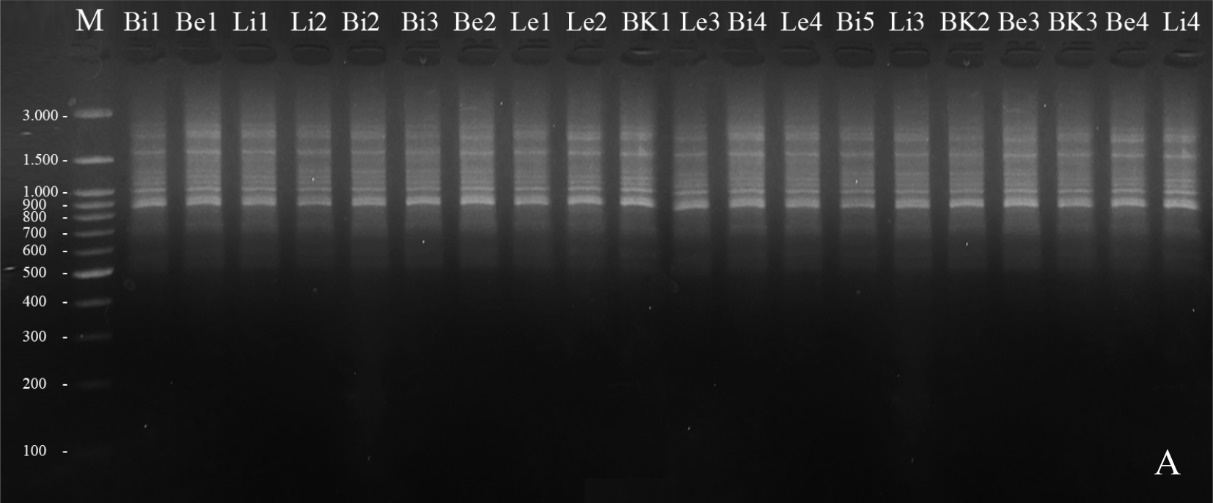


Figure S4: Electrophoresis results of 20 nutmeg samples from 4 sub-districts in Sangihe Island using UBC 807 primers. Notes: M: Geneaid 100 bp DNA Ladder marker. Bi: Thin round; Be: Thick round; Li: Thin oval; Le: Thick oval; BK: Twin seed.


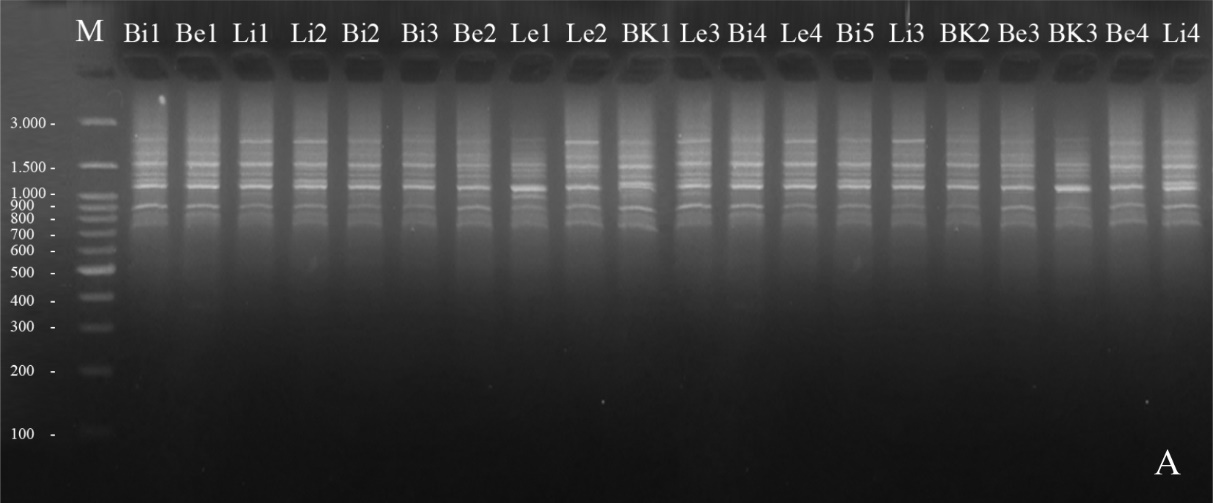


Figure S5: Electrophoresis results of 20 nutmeg samples from 4 sub-districts in Sangihe Island using UBC 434A primers. Notes: M: Geneaid 100 bp DNA Ladder marker. Bi: Thin round; Be: Thick round; Li: Thin oval; Le: Thick oval; BK: Twin seed.


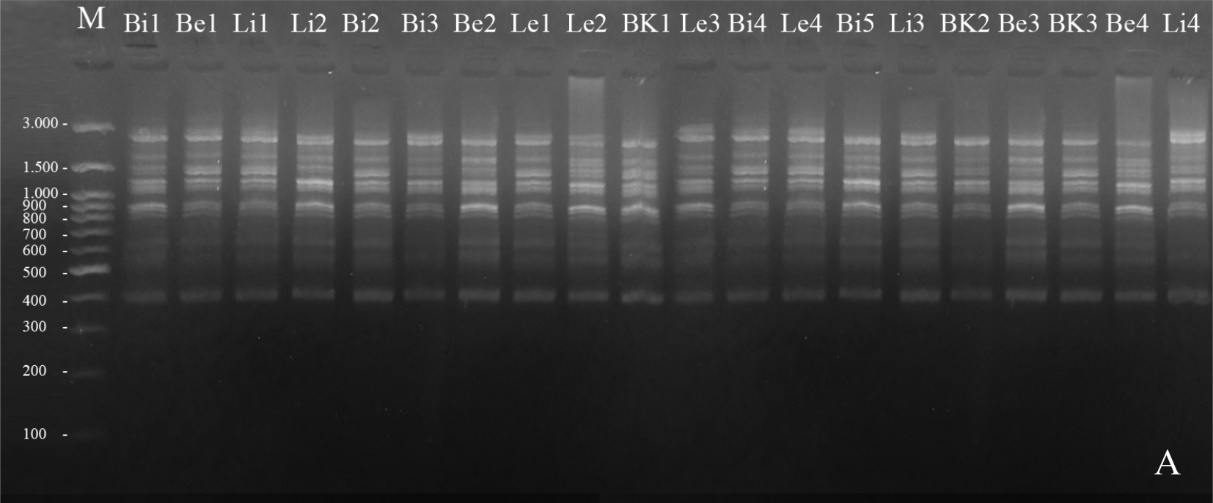


Figure S6: Electrophoresis results of 20 nutmeg samples from 4 sub-districts in Sangihe Island using UBC 840B primers. Notes: M: Geneaid 100 bp DNA Ladder marker. Bi: Thin round; Be: Thick round; Li: Thin oval; Le: Thick oval; BK: Twin seed.


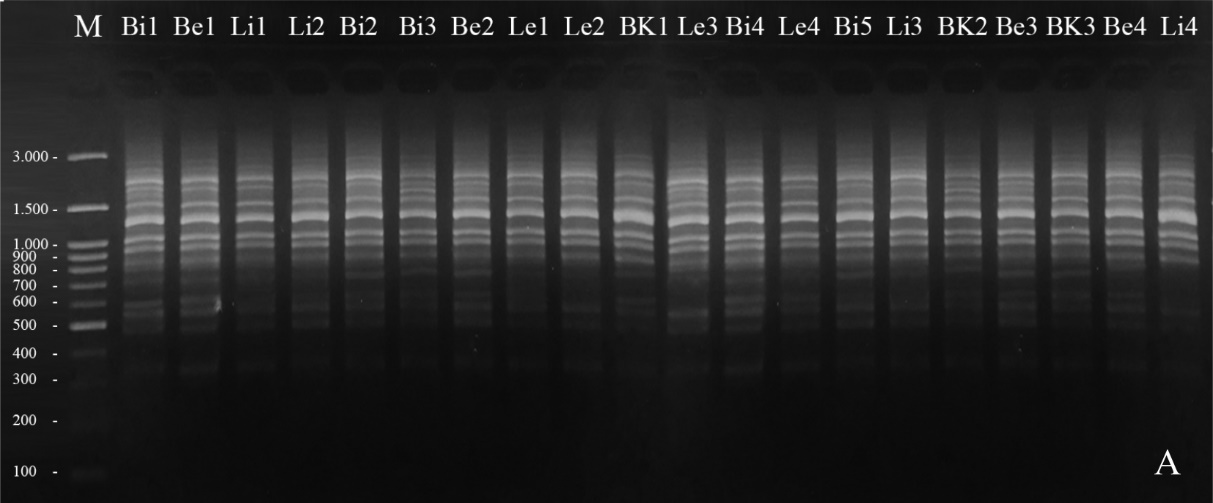


Figure S7: Electrophoresis results of 20 nutmeg samples from 4 sub-districts in Sangihe Island using UBC 842B primers. Notes: M: Geneaid 100 bp DNA Ladder marker. Bi: Thin round; Be: Thick round; Li: Thin oval; Le: Thick oval; BK: Twin seed.


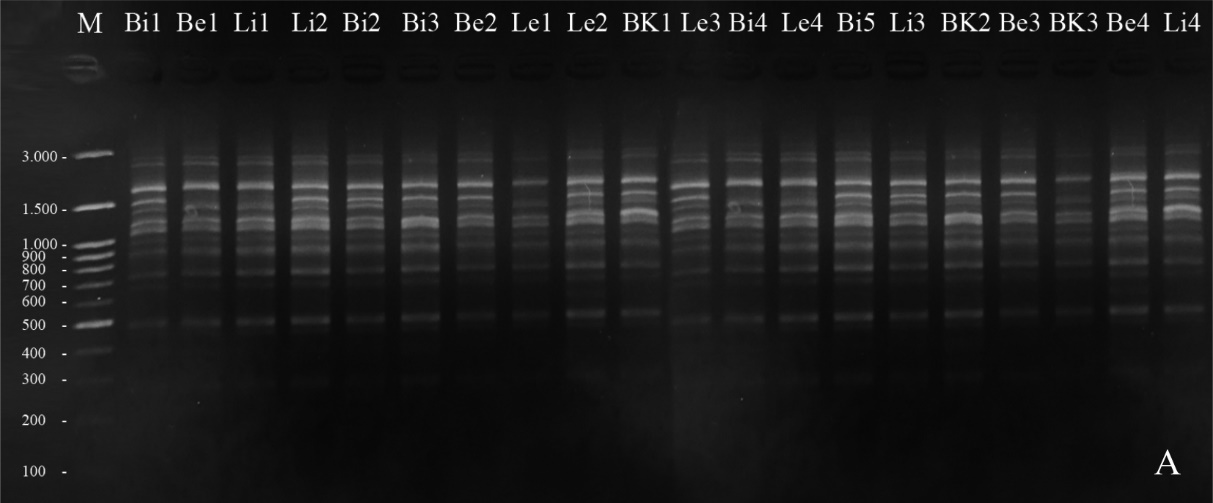


Figure S8: Electrophoresis results of 20 nutmeg samples from 4 sub-districts in Sangihe Island using UBC 810 primers. Notes: M: Geneaid 100 bp DNA Ladder marker. Bi: Thin round; Be: Thick round; Li: Thin oval; Le: Thick oval; BK: Twin seed.
